# Supplementary material for: Splice-Junction-Based Mapping of Alternative Isoforms in the Human Proteome
Source: Cell Rep. Author manuscript; Available in PMC 2020 Jan 15. (PMC6961840; doi:10.1016/j.celrep.2019.11.026)
Supplement: 2 [file NIHMS1546469-supplement-2.zip › DF1/PXD009737/TestisLysC/TestisLysC_26_RIN3_SLQPQAHSPRGDPTGPVPVVGKGEEEEEEDGMRLCLPANPK.pdf]

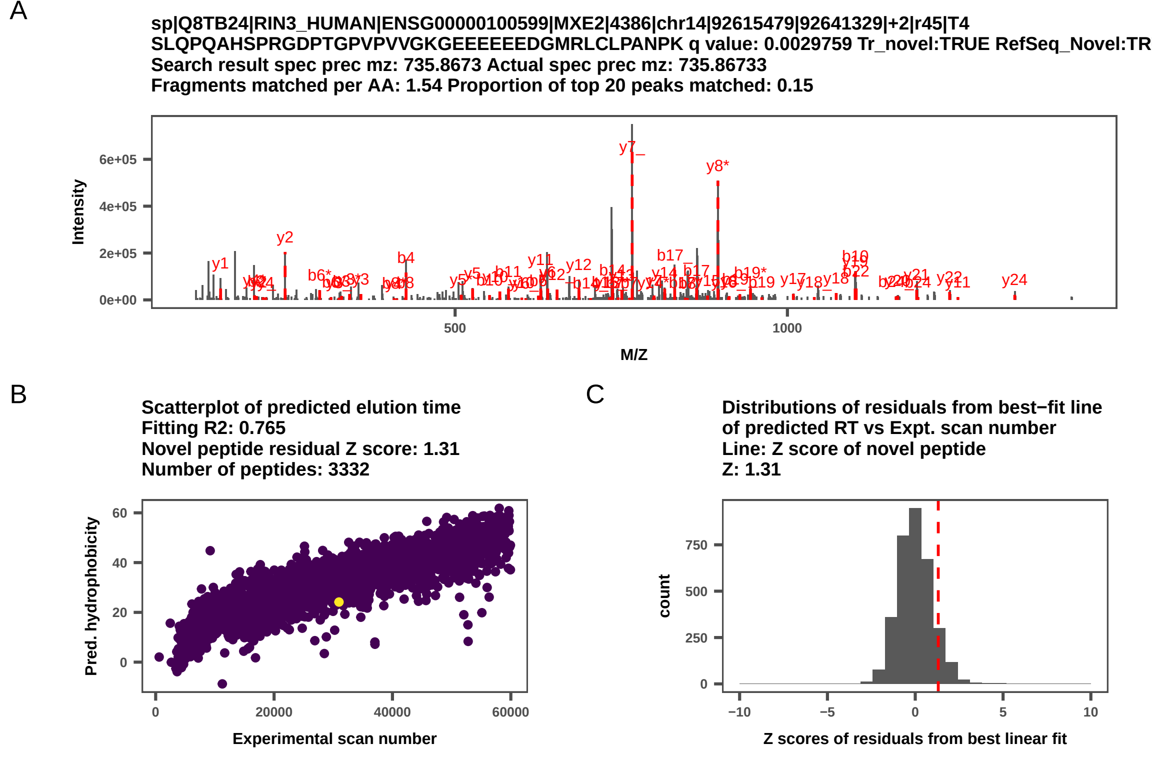
A
sp|Q8TB24|RIN3_HUMAN|ENSG00000100599|MXE2|4386|chr14|92615479|92641329|+2|r45|T4
SLQPQAHSPRGDPTGPVPVVGKGEEEEEEDGMRLCLPANPK q value: 0.0029759 Tr_novel:TRUE RefSeq_Novel:TR
Search result spec prec mz: 735.8673 Actual spec prec mz: 735.86733
Fragments matched per AA: 1.54 Proportion of top 20 peaks matched: 0.15
Intensity
6e+05
4e+05
2e+05
0e+00
y7_
y8*
y2
y1
b4
y11
y12
b17_
y24
500
1000
M/Z
B
Scatterplot of predicted elution time
Fitting R2: 0.765
Novel peptide residual Z score: 1.31
Number of peptides: 3332
Pred. hydrophobicity
60
40
20
0
0
20000
40000
60000
Experimental scan number
C
Distributions of residuals from best−fit line
of predicted RT vs Expt. scan number
Line: Z score of novel peptide
Z: 1.31
count
750
500
250
0
−10
−5
0
5
10
Z scores of residuals from best linear fit
